# The severity of andropause symptoms and its relationship with social well-being among retired male nurses: a preliminary cross-sectional study

**DOI:** 10.1186/s12877-024-04805-9

**Published:** 2024-02-23

**Authors:** Roya Nikjou, Mehdi Ajri-Khameslou, Shiva Jegargoosheh, Parisa Momeni, Reza Nemati-Vakilabad

**Affiliations:** 1 Department of Midwifery, School of Nursing and Midwifery, Ardabil University of Medical Sciences, Ardabil, Iran; 2 Department of Intensive Care Nursing, School of Nursing and Midwifery, Ardabil University of Medical Sciences, Ardabil, Iran; 3 Students Research Committee, School of Nursing and Midwifery, Ardabil University of Medical Sciences, Ardabil, Iran; 4 Student Research Committee, School of Nursing and Midwifery, Guilan University of Medical Sciences, Rasht, Iran

**Keywords:** Andropause, Testosterone, Retirement, Nurses, Social well-being

## Abstract

**Background:**

Andropause is a syndrome that occurs due to decreased androgen levels in men. Various aspects of health, such as social well-being, can affect andropause status during men’s retirement. This study aimed to determine the severity of andropause symptoms and its relationship with social well-being among retired male nurses.

**Methods:**

This preliminary cross-sectional study was conducted on 284 retired male nurses in Ardabil (northwest of Iran). The participants were selected through the census sampling method. Data were collected using a demographic information form, the Male Andropause Symptoms Self-Assessment Questionnaire (MASSQ), and the Social Well-Being Scale (SWBS). Data were analyzed using SPSS software (version 22.0).

**Results:**

The study found that the overall mean scores of the severity of andropause symptoms and social well-being among retired male nurses were 57.24 ± 12.62 (range = 35–91) and 94.54 ± 12.77 (range = 75–123), respectively. The highest and lowest mean scores between dimensions of social well-being were related to social contribution (20.26 ± 2.47) and social acceptance (15.26 ± 2.77), respectively. Multiple linear regression analysis revealed that subscales of social well-being, age, marital status, and spouse’s menopause were predictors of the severity of andropause symptoms among retired male nurses. The selected predictors accounted for 53.1% of the total variance in severity of andropause symptoms (F = 36.613, *p* < 0.001).

**Conclusion:**

The results showed a moderate to severe prevalence of andropause among retired male nurses and a significant association between andropause and social well-being. The study suggests further research to examine sexual orientation and other factors that may affect andropause in retired male nurses.

## Background

Nursing is a stressful job, and nurses are exposed to physical, mental, sexual, and social disorders due to exposure to various stresses such as high levels of work pressure, individual conflicts, rotational shifts, exposure to mortality, and lack of psychological support [1–3]. The National Association of Safety Professionals (NASP) has introduced nursing as one of the top 40 professions with a high severity of workplace-related disorders [4]. Evidence suggests that psychosocial stress related to the workplace may affect sexual function and androgen levels [5]. Kumar et al. [6] showed that the severity of sexual dysfunction was significantly higher in male nurses who endured stressful conditions in COVID-19-related wards than in those working in other departments. Pastuzak et al. [7] showed that men with non-standard working hours who do not have enough sleep and rest undergo decreased libido, increased hypogonadal symptoms, and sexual dysfunction. Güzel and Döndü [8] also reported that increased anxiety caused by the nursing work environment and a decrease in time spent with a spouse can cause sexual dysfunctions and even andropause in male nurses. Therefore, long-term work environment stress can affect different aspects of the social life of nurses and their family members and cause work-related family conflicts.

As a result of aging in men and physiological changes in the Hypothalamus-Pituitary-Gonadal (HPG) axis, plasma androgen levels decrease [9]. The gradual decrease in plasma testosterone level associated with symptoms of androgen deficiency is called andropause or Late-onset Hypogonadism (LOH) [10, 11]. Testosterone levels drop by approximately 0.4–2.6% per year after age 40 [12]. Testosterone levels at age 75 reach less than half of their amount compared to a healthy man between the ages of 20 and 30 [13, 14]. The severity of andropause varies among men in different parts of the world. Goel et al. [15] found the severity of andropause symptoms to be 11.6% in Indian men, and Araujo et al. [16] reported a severity rate of 5.6% in Spanish men. Khosravi et al. [17] showed the severity of andropause symptoms was 51.5% in Iranian men, and 3.5% of participants suffered from severe symptoms. The severity of andropause symptoms among Iranian nurses, especially retired nurses, has been less investigated. In a study conducted in northern Iran, the severity of sexual dysfunction in emergency male nurses was 40% [2].

Andropause is usually a hidden threat to men’s lives after the age of 40, and its clinical manifestations and symptoms are initially asymptomatic [18]. However, it is somewhat difficult to determine the specific range at which testosterone levels reach below the defined threshold level [19]. In general, as a result of lower testosterone levels, men may experience a range of symptoms, including decreased physical strength, weight gain, depressed mood, concentration disorder, decreased libido, muscle atrophy, insomnia, and hot flashes [20–22]. Although most of these symptoms are non-specific, their severity may vary in different people. Among the side effects of decreasing testosterone due to aging are the risk of metabolic diseases, diabetes mellitus, cardiovascular diseases, osteoporosis, hypertension, hyperlipidemia, decreased physical performance, and kidney diseases [22].

Trinick et al. [23] indicated a significant number of men suffer from hypogonadism, which does not have a definite diagnosis or treatment in scientific sources. Despite the importance of early diagnosis and treatment, several factors, such as the social environment and various aspects of health, such as mental health and social well-being, can negatively affect andropause in men during retirement [24–26]. Tan et al. stated the social environment can have a significant effect on men’s experiences of andropause. A supportive social environment can help men cope with the physical and emotional changes associated with andropause. In contrast, social norms, social isolation, and social stigma can exacerbate the adverse effects of this condition [27].

Social well-being (SWB) is considered one of the most important aspects of health in people’s lives [28]. SWB is one’s perceptions and experiences in social situations and their successful response to social challenges [29]. Physical, mental, emotional, and physiological disorders are less common in people with a high level of SWB [30–32]. Omidi and Rad [33] showed a significant correlation between SWB and marital adjustment. Therefore, various factors such as socioeconomic status, type of job, work environment, age, and social support can affect SWB [34, 35]. Cho et al. [36] showed that the hospital environment can influence health. Taheri et al. [37] reported the SWB status of Iranian healthcare providers, especially nurses, was moderate.

Andropause is a complex issue that is affected by several risk factors. Therefore, studies on the social dimensions determining andropause in Iran are insufficient. Previous studies have also considered the necessity of assessing nurses’ health status during the transition to retirement due to challenging factors [38, 39]. Therefore, conducting research such as this study to determine the severity of andropause symptoms in retired male nurses and its correlation with social well-being can play a role in filling the gap in the literature.

## Methods

### Design and participants

A preliminary cross-sectional study was conducted in Ardabil province in northwest Iran, following the Strengthening the Reporting of Observational Studies in Epidemiology (STROBE) guidelines [40].

The inclusion criteria were having no psychiatric disorders, taking no psychotropic medications (as self-reported by the participants), and retiring from one of the hospitals affiliated with Ardabil University of Medical Sciences. The exclusion criteria were lack of inclination to participate in the study, incomplete filling of questionnaires, and employment in private centers.

### Data collection

Data were collected from March to May 2022 using a census sampling method. The researchers went to Ardabil’s retirement centers. After stating the purpose of the study and obtaining permission from the relevant officials, they received the information of the retired nursing men (phone number, address, and Email). Based on this, telephone calls were made to establish an initial agreement (according to a similar protocol) with each selected individual. Then, after explaining the purpose of the study and obtaining informed consent, the questionnaire link was sent to each participant privately. So, all of them (309 people) agreed to participate in the study.

Data were collected using a demographic information form (including age, marital status, education level, underlying diseases, spouse’s menopause, and the number of children) and the Male Andropause Symptoms Self-Assessment Questionnaire (MASSQ) and Social Well-Being Scale (SWBS). The questionnaires were designed with web-based software, sent to the participants via Email and virtual networks (e.g., WhatsApp), and then completed and self-administered. A total of 284 participants completed the questionnaires. The response rate to the questionnaires was 91%.

#### Male andropause symptoms self-assessment questionnaire (MASSQ)

The MASSQ consisted of 25 items [41]. The items are rated on a five-point Likert scale. Each item was given a score from 1 (none) to 5 (severe). The overall scores of this scale ranged from 25 to 125, indicating no symptom to most severe symptom, respectively. The validity and reliability of this questionnaire among the Iranian population were assessed by Asadollahi et al. [41], with Cronbach’s alpha of 0.89 for the whole scale. The Cronbach’s alpha of the questionnaire in this study was calculated to be 0.81.

#### Social well-being scale (SWBS)

The Keyes SWBS (1998) [42] consisted of 33 items and five subscales, including social contribution (6 items), social cohesion (6 items), social acceptance (7 items), social integration (7 items), and social actualization (7 items). The questionnaire was scored on a 5-point Likert scale ranging from completely disagree (1) to completely agree (5). The range of scores obtained from this scale was 33 to 165, and higher scores indicated higher social well-being. The validity and reliability of the Persian version of this scale among the Iranian population were assessed by Nemati et al. [43], which showed satisfactory internal consistency (0.84) using Cronbach’s alpha. The Cronbach’s alpha of the scale was calculated to be 0.85 for this study.

### Data analysis

The data were analyzed using SPSS software version 22.0 (SPSS Inc., Chicago, IL, USA) using descriptive statistics (frequency, percentage, mean, and standard deviation). To investigate the relationship between participants’ andropause status, demographic variables, and social well-being, we used independent-sample t-test and Pearson’s correlation coefficient. Before performing the tests, the normality of the data distribution was checked using the one-sample Kolmogorov-Smirnov test (*p* > 0.05). Andropause predictors were determined using the Enter method of multiple linear regression. It should be noted that the data were investigated for linearity relationship assumptions, multivariate normality, independence of residuals, and multicollinearity before using multiple linear regression. The level of significance was considered to be 0.05.

### Ethical considerations

The proposal of this study was approved by the ethics committee of Ardabil University of Medical Sciences with the ethics code IR.ARUMS.REC.1400.321. No intervention was done on the participants, and doing this study was not dangerous for them. This study observed ethical considerations such as obtaining informed consent from the participants, principles of confidentiality, anonymity, and confidentiality of information.

## Results

A total of 284 retired male nurses participated in this study. The mean (SD) age of the participants was 58.41 (3.76) years. Almost one-third of the participants had an underlying disease (*n* = 94, 33.1%), and the education level of most of them (*n* = 266, 93.7%) was bachelor’s degree. The demographic characteristics of the participants are summarized in Table 1.

**Table 1:** Demographic characteristics of the participants (*n* = 284)

Variable	Categories	Mean ± SD	
Age Number of children		58.41 ± 3.76 2.22 ± 0.88	
		**Percentage**	**No.**
Marital status	Single	21.1	60
	Married	78.9	224 266
Education level	Bachelor	93.7	
	Master	6.30	18 105
Spouse menopause	Yes	37.0	
	No	63.0	179
Underlying Disease	Yes	33.1	94
	No	66.9	190

Table 2 identifies the relationship between demographic characteristics and the severity of andropause symptoms. There was a significant statistical correlation between The severity of andropause symptoms and certain demographic variables, such as age (*r* = 0.123, *p* < 0.001), marital status (t = -1.910, *p* > 0.001), spouse’s menopause (t = -8.279, *p* < 0.001), and underlying diseases (t = -1.187, *p* = 0.046). The results revealed that older retired male nurses had higher severity of andropause symptoms, and single retired male nurses scored higher than married retired male nurses. Participants whose spouses had gone through menopause also had higher scores. In addition, people with underlying diseases also scored higher.

**Table 2:** Association between the participants’ characteristics and the severity of andropause symptoms (*n* = 284)

Variables	Mean ± SD	r/t-value	*p*-value
**Age**	57.24 ± 12.62	0.123^a^	< 0.001
**Number of children**	57.24 ± 12.62	-0.106^a^	0.076
**Marital status**		-1.910^b^	< 0.001
Single	58.04 ± 12.15		
Married	54.26 ± 13.94		
**Education level**		0.225^b^	0.635
Bachelor	57.15 ± 12.85		
Master	58.61 ± 8.64		
**Spouse’s menopause**		-8.279^b^	< 0.001
Yes	61.52 ± 11.25		
No	49.94 ± 11.45		
**Underlying Diseases**		-1.787^b^	0.046
Yes	58.38 ± 11.44		
No	54.93 ± 14.51		

***Note.*** Correlation is significant at the *p* < 0.05.

^a^ Results of Pearson’s correlation coefficient (r).

^b^ Results of the independent-sample *t*-test.

The mean scores for the MASSQ and SWBS as well as their subscales are presented in Table 3. The mean (SD) of SWB was 94.54 (12.77) and that of andropause status was 57.24 (12.62). Social contribution with a mean (SD) of 20.26 (2.47) and social acceptance with a mean (SD) of 15.26 (2.77) obtained the highest and the lowest scores, respectively (Table 3).

**Table 3:** Mean (SD) of social well-being and andropause status (*n* = 284)

Variable	Subscales	SD	Mean
**Social well-being**	Social integration	4.92	19.19
	Social coherence	2.69	20.03
	Social contribution	2.77	20.26
	Social actualization	4.64	19.81
	Social acceptance	2.47	15.26
	SWB total score	12.77	94.54
**Andropause status total score**		12.62	57.24

To explain the andropause status of the participants, the Enter method of multiple linear regression analysis was performed. The results showed that the participants’ andropause status was determined by age, marital status, spouse’s menopause, and subscales of SWB (social integration, social actualization, and social acceptance). These variables could predict 53.1% of the andropause variance (Table 4).

**Table 4:** Linear regression analysis coefficients to examine predictors of andropause (*n* = 284)

Predictors	Unstandardized Coefficients (B)	SE	Standardized Coefficients (Beta)	t	*p*-value	Confidence Interval 95%	
						Lower Bound	Upper Bound
Age	1.831	0.174	0.547	10.526	< 0.001	1.489	2.174
Marital status	8.778	1.493	0.284	5.879	< 0.001	5.839	11.717
Spouse’s menopause	18.845	1.564	0.722	12.046	< 0.001	15.765	21.925
Underlying diseases	-0.004	0.265	-0.001	-0.015	0.953	-0.525	0.517
Social integration	-0.741	0.131	-0.289	-5.667	< 0.001	-0.998	-0.484
Social coherence	-0.390	0.259	-0.083	-1.505	0.134	-0.120	0.900
Social contribution	-0.098	0.327	-0.022	-0.301	0.763	-0.545	0.742
Social actualization	-0.491	0.188	-0.180	-2.616	0.009	-0.121	0.860
Social acceptance	-1.660	0.279	-0.325	-5.956	< 0.001	-2.208	-1.111

R^2^ = 0.546, Adjusted R^2^ = 0.531, F = 36.613, *p* < 0.001

## Discussion

The nursing profession is one of the occupations that disrupts nurses’ life cycle due to high work pressure and may damage their sexual health after retirement. This study investigated the correlation between andropause status and social well-being in retired male nurses.

The severity of andropause symptoms in retired male nurses was moderate to severe, which is comparable to previous studies. In a survey conducted in the north of Iran by Samipoor et al. [19] using the Aging Male Symptoms (AMS) scale, 73.6% of men over 40 years (age range: 40 to 76) experienced andropause symptoms.

Using the Brief Sexual Function Inventory (BSFI), Mohammadian and Dolatshahi [44] showed that the severity of erectile dysfunction in Iranian men living in Tehran was 40.4%, premature ejaculation was 32.5%, and libido disorder was 10.6%. Ebrahimian et al. [2] also used the Men’s Erectile Performance Questionnaire and showed that the severity of sexual dysfunction in emergency male nurses was 40%. In addition, studies conducted in other countries showed that the severity of andropause symptoms using ADAM and AMS questionnaires was 75.3% in South Korea and 70.94% in Indonesia, respectively [45, 46].

The mean score of andropause symptoms in this study was 57.24 (12.62). The findings were similar to those of other studies conducted in Iran by Rezaei et al. [11] and Hakimi et al. [47] using the MASSQ questionnaire, with mean scores of 57.46 (17.56) and 53.7 (9.9), respectively. However, Çetin [48] in Turkey showed that the mean score of andropause symptoms based on the MASSQ questionnaire among men 40 to 70 was 42.33 (13.47). This finding indicates that the severity of andropause symptoms in retired male nurses is almost the same as other men in Iranian society. Perhaps the difference between the results of the studies is due to the different conditions of the research environment and the lower sample size (125 men) compared to this study (284 men). Hence, the current survey as a preliminary study showed that moderate degrees of andropause will become a critical concern among the participants because, if neglected, they may experience severe andropause in the years to come. Public awareness, especially awareness among retired male nurses about the signs and symptoms of andropause, will help patients manage their sexual health problems with more appropriate treatments.

The results of this study indicated moderate SWB among the participants, which was 94.54 (12.77), which is in line with previous studies. In the study of Mozaffari et al. [28] conducted on Iranian nurses, the SWB was moderate, equal to 105.45 (15.87). In a study conducted on military retirees, the social well-being of the study group was desirable [35], which is in line with the present study’s findings. Nevertheless, in the study conducted on Iranian caregivers, the mean score of SWB was 62.28 (11.22), and the SWB score of nurses as the largest subgroup was 62.02 (10.67) [37]. Farahaninia et al. [49] also reported an SWB score of 67.32 (8.96) for nurses. In addition, a study in China on elderly people aged 60 to 89 years old showed the mean SWB score was 44.07 (6.69) [50]. The difference between the SWB mean scores among the studies may be due to the use of the short form of SWBS and the participants’ sociocultural differences. Accordingly, Salehi et al. [51] showed a statistically significant relationship between the nurses’ psychosocial health and job performance. In fact, it can be inferred that people with high SWB can more successfully cope with the challenges of playing the main roles. Hence, they are likely to be able to participate more in the family and community and adapt more to social norms.

The current study’s findings indicated a significant relationship between andropause symptoms and age. The severity of andropause symptoms increased with age. This finding was consistent with the results of previous studies [19, 52–54]. The results of studies have shown that a gradual decline in serum testosterone levels after the age of 50 can affect sexual function and sexual satisfaction [55, 56]. However, this finding was different from the results of Khosravi and Samipoor et al. [57, 58]. Perhaps the conflicting findings were due to the difference in the participants’ occupations and the instruments used to assess andropause symptoms.

Marital status was one of the effective and predictive factors for the severity of andropause symptoms. Married men had fewer andropause symptoms than single men. The results of a study in Kuwait on men aged 40 years and older showed a significant relationship between men’s awareness of andropause symptoms and marital status [53]. In contrast, the findings of Afsharnia et al. [54] were not consistent with the findings of this study. Andropause symptoms in men may be influenced by sexual relationships and satisfaction with the spouse’s behaviors. Further studies are needed to increase the generalizability of these findings.

Participants’ andropause symptoms were significantly associated with their spouse’s menopause. Rezaei et al.‘s study results were also consistent with our findings [11]. Menopause in women is mainly associated with physical, mental, and sexual changes [59]. On the other hand, andropause in men may occur at the same time as their spouse’s menopause. The coincidence of andropause with menopause can be associated with a decrease in the quality of marital relationships and exacerbation of andropause symptoms in men [59], which confirms the findings of our study.

The linear regression model results showed that the underlying disease was not related to the participants’ andropause symptoms. The findings of previous studies were consistent with our results [2, 60]. A review also showed that testosterone replacement therapy does not affect cardiovascular diseases [61]. However, Rezaei et al. [11] showed that andropause scores were associated with depression, coronary heart disease, and urinary incontinence. Previous studies have indicated a relationship between decreased testosterone levels and chronic diseases such as hypertension, cardiovascular diseases, and mental disorders [17, 62, 63]. These differences seem to be due to the different research communities and their work stress level. Further experimental studies are required to investigate these contradictions more precisely.

The study results also showed an inverse relationship between andropause symptoms and SWB among retired male nurses. Comparing the results of this study with those of previous studies is somewhat difficult, as no studies have examined the relationship between andropause symptoms and the SWB status of retired nurses. According to a survey conducted in the United States on patients with brain tumors, the sexual function of patients was inversely correlated with their social well-being. The researchers attributed the finding to the type of brain tumor and its region [64]. A study conducted in South Korea on the health-related quality of life in prostate cancer patients receiving androgen deprivation therapy (ADT) showed the lowest mean score for the social well-being subscale. The researchers suggested that given the limited evidence, the effect of culture on the SWB of men receiving ADT should be specifically considered [65]. The findings of these two studies confirmed the results of the current study. It should be noted that the social integration, social actualization, and social acceptance subscales were the predictors of andropause symptoms, which is a new finding among retired male nurses. A study conducted in Indonesia on the relationship between sexual health and different aspects of health showed that social integration was one of the factors affecting sexual health [66].

As a preliminary study, these results suggest that more research is needed to fully understand the relationship between andropause symptoms and SWB among retired male nurses. Future studies are suggested to examine SWB and spouse’s menopause.

### Limitations

This research had some limitations. One of the limitations of this study was its cross-sectional nature, which constrained the interpretation of the causal relationship between the variables. Longitudinal and experimental studies are suggested to measure blood androgen levels to understand the causal relationship between the variables and explore more useful information about andropause. Furthermore, using a self-administered questionnaire to assess andropause symptoms may be another limitation of this study. Since andropause is an important issue, some participants may not have answered the questions accurately enough. Nevertheless, the current study’s design was based on the population of retired male nurses, and the participants were selected by census sampling method, which is considered its strength. Therefore, it is possible to generalize the results to the whole population of retired male nurses.

## Conclusion

In general, the findings of this study showed that SWB is an essential determinant of andropause symptoms in retired male nurses, and some background variables are effective in increasing the severity of andropause symptoms, including age, marital status, and spouse’s menopause. Accordingly, the relatively moderate to severe severity of andropause in the Iranian elderly population, especially retired nurses, highlights the necessity of improving social conditions. In other words, strategies affecting the promotion of SWB, especially in dimensions that predicted andropause symptoms, should be developed and implemented. Nursing managers can consider these results to improve the SWB of retired nurses by planning and providing the necessary measures. Hence, assessing the severity of andropause symptoms and the SWB status of retired male nurses in other regions can provide useful information. In addition, more studies are recommended for the paraclinical evaluation of andropause and the discovery of other factors affecting it in retired male nurses.

## Data Availability

The datasets used and/or analyzed during the current study are available from the corresponding author on reasonable request.

## Abbreviations

MASSQ: Male andropause symptoms self-assessment questionnaire
SWBS: Social well-being scale
SPSS: Statistical Package for the Social Sciences

## References

[1] Lambert VA, Lambert CE, Petrini M, Li XM, Zhang YJ (2007). Workplace and personal factors associated with physical and mental health in hospital nurses in China. Nurs Health Sci.

[2] Ebrahimian A, Hashemi-Amre S-H, Homami S, Fakhr-Movahedi A. Sexual Dysfunction and Related Factors in Hospitals Emergency Male Nurses and Its Relationship with Their Spouse’s Sexual Function. Middle East J Rehabilitation Health Stud 2022(In Press).

[3] Bilge C, Mecdi Kaydirak M, Gür Avci D, Hotun Sahin N (2020). Effect of Shift Working on Depression prevalence and sexual life of female nurses: a Correlational Study in Turkey. Int J Sex Health.

[4] Pazhouhan A (2016). The relationship between emotional intelligence and occupational stress among nurses of Alzahra Hospital in Isfahan. J Hosp.

[5] Mokarami H, Toderi S, Rahimi Pordanjani T, Taban E (2018). Role of psychosocial job stressors on sexual function of male nurses: the mediator role of work ability. Am J Men’s Health.

[6] Kumar M, Mohindra R, Sharma K, Soni RK, Rana K, Singh SM (2021). The impact of working in a COVID hospital on sexual functioning in male nurses: a study from North India. Ind Psychiatry J.

[7] Pastuszak AW, Moon YM, Scovell J, Badal J, Lamb DJ, Link RE, Lipshultz LI (2017). Poor sleep quality predicts hypogonadal symptoms and sexual dysfunction in male nonstandard shift workers. Urology.

[8] Güzel A, Döndü A. Changes in sexual functions and habits of healthcare workers during the ongoing COVID-19 outbreak: a cross-sectional survey study. Ir J Med Sci (1971-) 2021:1–9. 10.1007/s11845-021-02691-3 PMC8244671 34195918

[9] Martelli M, Zingaretti L, Salvio G, Bracci M, Santarelli L (2021). Influence of work on andropause and menopause: a systematic review. Int J Environ Res Public Health.

[10] Abootalebi M, Kargar M, Aminsharifi A (2017). Assessment of the validity and reliability of a questionnaire on knowledge and attitude of general practitioners about andropause. Aging Male.

[11] Rezaei N, Azadi A, Pakzad R (2020). Prevalence of andropause among Iranian men and its relationship with quality of life. Aging Male.

[12] Liu C-C, Wu W-J, Lee Y-C, Wang C-J, Ke H-L, Li W-M, Hsiao H-L, Yeh H-C, Li C-C, Chou Y-H (2009). The prevalence of and risk factors for androgen deficiency in aging Taiwanese men. J Sex Med.

[13] Staerman F, Léon P (2012). Andropause (androgen deficiency of the aging male): diagnosis and management. Minerva Med.

[14] Hackney AC. Hypogonadism in exercising males: Dysfunction or Adaptive-Regulatory Adjustment? Front Endocrinol 2020, 11. 10.3389/fendo.2020.00011 PMC7005256 32082255

[15] Goel A, Sinha RJ, Dalela D, Sankhwar S, Singh V (2009). Andropause in Indian men: a preliminary cross-sectional study. Urol J.

[16] Araujo AB, Esche GR, Kupelian V, O’Donnell AB, Travison TG, Williams RE, Clark RV, McKinlay JB (2007). Prevalence of symptomatic androgen deficiency in men. J Clin Endocrinol Metabolism.

[17] Khosravi S, Ardebili HE, Larijani B, Nedjat S, Nikbakht Nasrabadi A, Ardebili ME, Dabiran S, Samizadeh E (2015). Are andropause symptoms related to depression?. Aging Clin Exp Res.

[18] Jannini EA, Nappi RE (2018). Couplepause: a new paradigm in treating sexual dysfunction during menopause and andropause. Sex Med Reviews.

[19] Samipoor F, Pakseresht S, Rezasoltani P, Kazemnajad Leili E (2017). Awareness and experience of andropause symptoms in men referring to health centers: a cross-sectional study in Iran. Aging Male.

[20] Zhang Z, Qiu S, Huang X, Jin K, Zhou X, Lin T, Zou X, Yang Q, Yang L, Wei Q (2022). Association between testosterone and serum soluble α-klotho in US males: a cross-sectional study. BMC Geriatr.

[21] Shigehara K, Konaka H, Koh E, Izumi K, Kitagawa Y, Mizokami A, Nakashima T, Shimamura M, Iwamoto T, Namiki M (2015). Effects of testosterone replacement therapy on nocturia and quality of life in men with hypogonadism: a subanalysis of a previous prospective randomized controlled study in Japan. Aging Male.

[22] Afsharnia E, Pakgohar M, Khosravi S, Haghani H (2016). The quality of life and related factors in men with andropause. Hayat.

[23] Trinick TR, Feneley MR, Welford H, Carruthers M (2011). International web survey shows high prevalence of symptomatic testosterone deficiency in men. Aging male.

[24] Lambrinoudaki I, Armeni E, Goulis D, Bretz S, Ceausu I, Durmusoglu F, Erkkola R, Fistonic I, Gambacciani M, Geukes M (2022). Menopause, wellbeing and health: a care pathway from the European menopause and Andropause Society. Maturitas.

[25] Kim ES, Tkatch R, Martin D, MacLeod S, Sandy L, Yeh C (2021). Resilient aging: Psychological Well-Being and Social Well-being as targets for the Promotion of healthy aging. Gerontol Geriatr Med.

[26] Kim S, Lee S-H, Hong E (2020). Effects of Andropause Syndrome, Resilience on Retirement anxiety in Middle-aged men. J Industrial Convergence.

[27] Tan R, Philip P (1999). Perceptions of and risk factors for andropause. Arch Androl.

[28] Mozaffari N, Dadkhah B, Shamshiri M, Mohammadi MA, Nayeri ND (2014). The status of social well-being in Iranian nurses: a cross-sectional study. J Caring Sci.

[29] Javadi-Pashaki N, Darvishpour A (2018). What are the predictor variables of social well-being among the medical science students?. J Educ Health Promot.

[30] Khamisa N, Oldenburg B, Peltzer K, Ilic D (2015). Work related stress, burnout, job satisfaction and General Health of Nurses. Int J Environ Res Public Health.

[31] Caddick N, Smith B (2014). The impact of sport and physical activity on the well-being of combat veterans: a systematic review. Psychol Sport Exerc.

[32] Edwards S (2006). Physical exercise and psychological well-being. South Afr J Psychol.

[33] Omidi R, Rad F (2019). The relationship between Social health and marital Adjustment among married women in Astara. Sociol Stud.

[34] Lee KH, Yoon DP (2011). Factors influencing the general well-being of low-income Korean immigrant elders. Soc Work.

[35] Afshar PF, Foroughan M, Pirooz F, Ajri M (2020). Social well-being of Iranian retired men of the armed forces and their wives. BMJ Military Health.

[36] Cho H, Han K (2018). Associations among nursing work environment and health-promoting behaviors of nurses and nursing performance quality: a multilevel modeling approach. J Nurs Scholarsh.

[37] Taheri M, Ghasemi E, Negarandeh R, Janani L, Mirbazegh F (2019). Social Wellbeing among Iranian caregivers. Soc Indic Res.

[38] Feng J, Li Q, Smith JP (2020). Retirement effect on health status and health behaviors in urban China. World Dev.

[39] Gabrielle S, Jackson D, Mannix J (2008). Older women nurses: health, ageing concerns and self-care strategies. J Adv Nurs.

[40] Von Elm E, Altman DG, Egger M, Pocock SJ, Gøtzsche PC, Vandenbroucke JP, Initiative S (2014). The strengthening the reporting of Observational studies in Epidemiology (STROBE) Statement: guidelines for reporting observational studies. Int J Surg.

[41] Asadollahi A, Saberi LF, Faraji N (2013). Validity and reliability of male andropause symptoms self-assessment questionnaire among elderly males in Khuzestan province of Iran. J mid-life Health.

[42] Keyes CLM. Social well-being. Social Psychol Q 1998:121–40.

[43] Vakilabad RN, Kheiri R, Islamzadeh N, Afshar PF, Ajri-Khameslou M (2023). A survey of social well-being among employees, retirees, and nursing students: a descriptive-analytical study. BMC Nurs.

[44] Mohammadian S, Dolatshahi B (2019). Sexual problems in Tehran: prevalence and associated factors. J Educ Health Promot.

[45] Kang S, Park HJ, Park NC (2013). Serum total testosterone level and identification of late-onset hypogonadism: a community-based study. Korean J Urol.

[46] Taher A (2005). Proportion and acceptance of andropause symptoms among elderly men: a study in Jakarta. Acta Med Indones.

[47] Hakimi S, Ghasemi L, Mirghafourvand M, Hassanzadeh K, Ghasemi F (2019). The prevalence of andropause symptoms and the role of social determinants of health on its severity in healthy men: a community-based study in Northwest Iran. Crescent J Med Biol Sci.

[48] Çetin AM. Andropoz Belirtileri Öz Değerlendirme Ölçeğinin Türkçe Geçerlik ve Güvenirliğinin Araştırılması ve Yaşlanmaya Bağlı Gelişen Andropozun Fiziksel Aktivite ve Yaşam Kalitesi ile İlişkisinin İncelenmesi. 2021.

[49] Farahaninia M, Ehyaei P, Ahmadi Z, Haghani H (2019). Relationship between nurses’ Social Health and Quality of Life. J Client-Centered Nurs Care.

[50] Du X, Zhou M, Mao Q, Luo Y, Chen X. Positive aging: social support and social well-being in older adults-the serial mediation model of social comparison and cognitive reappraisal. Curr Psychol 2022.

[51] Salehi A, Marzban M, Sourosh M, Sharif F, Nejabat M, Imanieh MH (2017). Social well-being and related factors in students of school of nursing and midwifery. Int J Community Based Nurs Midwifery.

[52] Chen RY-t, Ng K-K (2010). Self-referred older Asian males in a men’s health clinic: the inter-relationships between androgens, metabolic parameters and quality of life measures. Aging Male.

[53] Maha A-S (2013). Prevalence of andropausal symptoms among Kuwaiti males. Am J Men’s Health.

[54] Afsharnia E, Pakgohar M, Haghani H, Sarani A, Khosravi S (2020). The severity of hypogonadism symptoms and its risk factors among male employees of Tehran University of Medical Sciences. Aging Male.

[55] Shin YS, You JH, Cha JS, Park JK (2016). The relationship between serum total testosterone and free testosterone levels with serum hemoglobin and hematocrit levels: a study in 1221 men. Aging Male.

[56] Rezanezhad B, Borgquist R, Willenheimer R, Elzanaty S (2018). Association between serum levels of testosterone and biomarkers of subclinical atherosclerosis. Aging Male.

[57] Samipoor F, Pakseresht S, Rezasoltani P, Mehrdad M (2018). The association between hypogonadism symptoms with serum testosterone, FSH and LH in men. Aging Male.

[58] Khosravi S. Explanation of life experience andropause in men and offering cultural-base health promotion’s intervention program and evaluation. *Tehran: Tehran University of Medical Sciences and Health Services* 2015.

[59] Nisar N, Sohoo NA (2010). Severity of menopausal symptoms and the quality of life at different status of menopause: a community based survey from rural Sindh, Pakistan. Int J Collaborative Res Intern Med Public Health.

[60] Omar SM, Musa IR, Idrees MB, Abdelbagi O, Adam I (2022). Prevalence and associated factors of erectile dysfunction in men with type 2 diabetes mellitus in eastern Sudan. BMC Endocr Disorders.

[61] Schwarz ER, Phan A, Willix RD (2011). Andropause and the development of cardiovascular disease presentation—more than an epi-phenomenon. J Geriatric Cardiology: JGC.

[62] Gökçe M, Yaman Ö (2017). Erectile dysfunction in the elderly male. Turk J Urol.

[63] Kulej-Lyko K, Majda J, von Haehling S, Doehner W, Lopuszanska M, Szklarska A, Banasiak W, Anker SD, Ponikowski P, Jankowska EA (2016). Could gonadal and adrenal androgen deficiencies contribute to the depressive symptoms in men with systolic heart failure?. Aging Male.

[64] Boccia ML, Anyanda EI, Fonkem E (2021). A preliminary report on quality of life and sexual function in brain tumor patients. J Sex Med.

[65] Kim SH, Yoon SM, Choi YD, Choi E, Song H (2017). Predictors of health-related quality of life in Korean prostate cancer patients receiving androgen deprivation therapy. Eur J Oncol Nurs.

[66] Hensel DJ, Nance J, Fortenberry JD (2016). The association between sexual health and physical, mental, and social health in adolescent women. J Adolesc Health.

